# Mice with Type 2 Diabetes Present Significant Alterations in Their Tissue Biomechanical Properties and Histological Features

**DOI:** 10.3390/biomedicines10010057

**Published:** 2021-12-28

**Authors:** Tânia B. Cruz, Filomena A. Carvalho, Paulo N. Matafome, Raquel A. Soares, Nuno C. Santos, Rui D. Travasso, Maria J. Oliveira

**Affiliations:** 1 i3S, Institute for Research and Innovation in Health, University of Porto, Rua Alfredo Allen, 4200-135 Porto, Portugal; raqsoa@med.up.pt (R.A.S.); mariajo@ineb.up.pt (M.J.O.); 2 INEB, National Institute of Biomedical Engineering, Rua Alfredo Allen, 4200-135 Porto, Portugal; 3 Instituto de Medicina Molecular, Faculdade de Medicina, Universidade de Lisboa, Avenida Prof. Egas Moniz, 1649-028 Lisbon, Portugal; filomenacarvalho@medicina.ulisboa.pt (F.A.C.); nsantos@fm.ul.pt (N.C.S.); 4 iCBR, Coimbra Institute for Clinical and Biomedical Research, Faculty of Medicine, University of Coimbra, Azinhaga Santa Comba, 3000-548 Coimbra, Portugal; paulo.matafome@uc.pt; 5 CIBB, Center for Innovative Biomedicine and Biotechnology, University of Coimbra, Rua Larga, 3004-516 Coimbra, Portugal; 6 CACC, Clinical-Academic Center of Coimbra, Faculty of Medicine, University of Coimbra, Rua Larga, 3004-516 Coimbra, Portugal; 7 Department of Biomedicine, Faculty of Medicine, University of Porto, Alameda Prof. Hêrnani Monteiro, 4200-319 Porto, Portugal; 8 CFisUC, Centre for Physics of the University of Coimbra, Department of Physics, Rua Larga, 3004-516 Coimbra, Portugal; ruit@uc.pt; 9 ICBAS, Institute of Biomedical Sciences Abel Salazar, University of Porto, Rua Jorge de Viterbo Ferreira, 4050-313 Porto, Portugal

**Keywords:** extracellular matrix biomechanical properties, tissue decellularization, ECM component distribution, diabetes-associated ECM modifications

## Abstract

Type 2 diabetes mellitus (T2DM) is a complex metabolic disease often associated with severe complications that may result in patient morbidity or death. One T2DM etiological agent is chronic hyperglycemia, a condition that induces damaging biological processes, including impactful extracellular matrix (ECM) modifications, such as matrix components accumulation. The latter alters ECM stiffness, triggering fibrosis, inflammation, and pathological angiogenesis. Hence, studying ECM biochemistry and biomechanics in the context of T2DM, or obesity, is highly relevant. With this in mind, we examined both native and decellularized tissues of obese B6.Cg-Lep^ob^/J (ob/ob) and diabetic BKS.Cg-Dock7m+/+Lepr^db^J (db/db) mice models, and extensively investigated their histological and biomechanical properties. The tissues analyzed herein were those strongly affected by diabetes—skin, kidney, adipose tissue, liver, and heart. The referred organs and tissues were collected from 8-week-old animals and submitted to classical histological staining, immunofluorescence, scanning electron microscopy, rheology, and atomic force microscopy. Altogether, this systematic characterization has identified significant differences in the architecture of both ob/ob and db/db tissues, namely db/db skin presents loose epidermis and altered dermis structure, the kidneys have clear glomerulopathy traits, and the liver exhibits severe steatosis. The distribution of ECM proteins also pinpoints important differences, such as laminin accumulation in db/db kidneys and decreased hyaluronic acid in hepatocyte cytoplasm in both obese and diabetic mice. In addition, we gathered a significant set of data showing that ECM features are maintained after decellularization, making these matrices excellent biomimetic scaffolds for 3D in vitro approaches. Importantly, mechanical studies revealed striking differences between tissue ECM stiffness of control (C57BL/6J), obese, and diabetic mice. Notably, we have unveiled that the intraperitoneal adipose tissue of diabetic animals is significantly stiffer (G* ≈ 10,000 Pa) than that of ob/ob or C57BL/6J mice (G* ≈ 3000–5000 Pa). Importantly, this study demonstrates that diabetes and obesity selectively potentiate severe histological and biomechanical alterations in different matrices that may impact vital processes, such as angiogenesis, wound healing, and inflammation.

## 1. Introduction

Type 2 diabetes mellitus (T2DM) is a chronic metabolic disorder that affects more than 300 million individuals worldwide. This pathologic condition is associated with multiple disabling complications that ultimately may lead to death. T2DM patients, often obese, suffer from cardiovascular deficiencies, nephropathies, retinopathies, neuropathies, and skin ulcers, such as diabetic foot. The etiology of this metabolic disease is insulin resistance, a condition where cells fail to respond to normal insulin levels, initially leading to hyperinsulinemia to maintain normal glucose levels. Yet, the sustained production of elevated insulin levels results in the exhaustion of pancreatic β cells ability to produce insulin and leads to chronic hyperglycemia [1]. It is accepted that obesity and the continuous exposure to high glucose levels are two of the main factors leading to the development of T2DM complications [2,3]. The persistence of elevated glucose levels in the organism leads to the glycation of different molecules and mediates the formation of advanced glycation end (AGE) products that may covalently crosslink and, biochemically, reshape the structure and function of numerous proteins [4,5]. AGE-modified precursors also inflict alterations to extracellular matrix (ECM) fibrous proteins, such as collagen, elastin, fibronectin, and laminin, promoting matrix accumulation and changes in stiffness, thus affecting tissue functions [6]. Furthermore, AGE products can bind to the receptor for AGE (RAGE) triggering fibrosis, inflammation, and pathological angiogenesis [2,5,6,7,8]. As such, it is clear the existence of an intricate relationship between hyperglycemia and ECM deposition and remodeling [9]. Nevertheless, little is known about the impact of diabetes and, in particular, of high glucose levels in the ECM biomechanical properties, a complex and dynamic fiber network that surrounds cells and modulates their activity. So far, it was reported that plantar skin from diabetic patients, highly prone to ulcerations, presents increased stiffness when compared to that of non-diabetic controls. In these patients, the Young’s Modulus from the tissue bellow the calcaneus differs from those of other plantar areas, having the lowest stiffness of the studied foot regions [10]. In line with this, Boivin et al. used a diabetic mouse model to study the Achilles tendon, a structure of the foot that is often injured when a diabetic foot ulcer develops. The Achilles tendon biomechanical and histological evaluation unveiled that, although the diabetic tendon had a higher diameter, it presented a significant decrease in stiffness, and elastic modulus when compared to controls [11]. Other studies revealed alterations in the diabetic heart biomechanical properties, such as increased diastolic stiffness, excessive diastolic left ventricular rigidity, and myocyte increased Young’s Modulus, important contributors to heart failure in T2DM patients [12,13]. Nevertheless, a common limitation of these studies is the use of native tissues to address the biomechanical attributes of the diabetic ECM. Such approach does not allow the exclusion of cell mechanics over the matrix. To overcome this constraint and investigate both ECM histological and biomechanical properties, we used decellularized tissues from animal models of obesity and of T2DM, BKS.Cg-Dock7m+/+Lepr^db^J (db/db) mice, and compared them with the acellular tissues from obese non-diabetic B6.Cg-Lep^ob^/J (ob/ob) animals and with C57BL/6J controls. We fully characterized the main T2DM affected organs and tissues, namely skin, kidney, liver, heart, and adipose tissue from the referred db/db and ob/ob mice, detailing their histology, architecture, and ECM component patterns. We identified important differences, particularly in the skin, kidney, and liver of db/db animals when compared to C57BL/6J controls. Immunofluorescence against ECM proteins is suggestive of differences between diabetic and non-diabetic rodents and indicate that decellularized tissues maintain the ECM components, although their patterns may differ from those of the native counterparts. Thus, these scaffolds are undoubtedly valuable models to assess the biomechanical properties of obese T2DM bearing mice and of obese non-diabetic animals ECM. Importantly, we show that diabetic matrices, when compared with normal (C57BL/6J) or obese (ob/ob), exhibit significantly different stiffness and viscoelastic properties. Altogether, the results gathered pinpoint relevant T2DM-associated alterations in matrix structural, biological, and mechanical properties potentially impactful to the disease pathophysiology.

## 2. Materials and Methods

### 2.1. Ethics Statement

All experimental animal procedures were approved by the Animal Ethics Committee of the Institute for Research & Innovation in Health (i3S), University of Porto, Portugal, and licensed by DGAV (General Directory of Food and Veterinary, Ministry of Agriculture, Rural Development and Fishing, Government of Portugal). All animals were handled in strict accordance with good animal practice as defined by national authorities (DGAV, Law nu1005/92 from 23 October 1992) and European Parliament Directive 2010/63/EU.

### 2.2. Animals and Organ Collection

Heterozygous obese, B6.Cg-Lep^ob^/J (ob/ob), and diabetic, BKS.Cg-Dock7^m^+/+Lepr^db^J (db/db), animals were purchased from Charles River, Madrid, Spain, and bred at the i3S animal house under specific pathogen-free conditions. All animals were kept in an ad libitum standard diet (2014S Teklad Diets) and with free access to sterilized tap water. Each litter was closely monitored and genotyped when a new mating had been required. Homozygous −/− C57BL/6J (wild type) mice from the same litters of either ob/ob or db/db transgenic strains were used as respective controls. Six-week-old animals were weighted periodically and monitored for blood glucose levels (non-fasting). Eight-week-old obese (ob/ob), diabetic (db/db), and C57BL/6J (control) male mice were euthanized with carbon dioxide followed by necropsy to collect skin, adipose tissue, kidneys, liver, and heart. The organs and the intraperitoneal adipose tissue were cleaned with HBSS (Gibco, Waltham, MA, USA), embedded in OCT (Thermo Scientific, Waltham, MA, USA), cryopreserved in liquid nitrogen cooled isopentane (Sigma-Aldrich, St. Louis, MO, USA), and immediately stored at −80 °C. For body fat quantification, all adipose tissue depots were carefully removed, washed with saline, blotted-dried, and weighted.

### 2.3. Decellularization

Murine samples, previously frozen, were washed in PBS, sliced with the help of a 4 × 4 mm^2^ grid (thickness of approximately 2 to 4 mm), and placed in a 24-well plate. Decellularization was achieved through a sequence of incubation steps as described elsewhere [14,15]. Briefly, tissue fragments were incubated in a hypotonic buffer (10 mM Tris-HCl, 0.1% EDTA, pH 7.8) for 18 h, washed with PBS, and further incubated in a buffered solution containing sodium dodecyl sulfate (SDS) at 0.1% for liver and adipose tissue, 0.2% for skin and kidney, or 0.3% for heart samples. After 24 h, fragments were washed in 10 mM Tris-HCl pH 7.8, treated with 50 U/mL DNase I (AppliChem GmbH, Darmstadt, Germany) for 3 h at 37 °C, and washed in PBS. Decellularized matrices were stored at 4 °C in PBS supplemented with 10 mg/mL gentamicin (Gibco) and 1% fungizone (Alfagene, Lisbon, Portugal), for a maximum period of four weeks. Of note, a small fragment of each tissue, from each animal, was kept as a “Native” sample for further comparison.

### 2.4. DNA Quantification

Total DNA content was assessed in native and decellularized tissues from ob/ob, db/db, and C57BL/6J control animals. For that, DNA was extracted using PureLink Genomic DNA Mini Kit (Invitrogen, Waltham, MO, USA), according to the manufacturer’s instructions, and quantified in a NanoDrop 1000, as ng of DNA per mg of tissue.

### 2.5. Histology and Immunofluorescence Assays

Native and decellularized tissues from ob/ob, db/db, and C57BL/6J controls were formalin-fixed, paraffin-embedded, and sliced into 3 µm-thick sections. For histology, each sample was stained with Hematoxylin and Eosin (H&E) and Masson’s Trichrome. A light microscope Olympus CX31 coupled with a DP-25 Camera was used to acquire all histology images. For immunofluorescence, the sections were dewaxed in xylene, rehydrated with a graded ethanol-water series, and subjected to antigen retrieval by heat-induced epitope retrieval. Tissue sections were then incubated with 1% bovine serum albumin (Sigma-Aldrich) at room temperature (RT) for 2 h, followed by incubation with the primary antibody overnight at 4 °C. Antibodies directed to major structural ECM components such as fibronectin (1:400 rabbit polyclonal, F3648, Sigma-Aldrich), laminin (1:100 rabbit polyclonal, L9393, Sigma-Aldrich), collagen IV (1:10 rabbit polyclonal, AB769, Millipore, Burlington, MA, USA), and hyaluronic acid binding protein (1:200, 385911, Millipore) were used. The incubation with secondary antibodies was performed at RT for 1 h, using goat anti-rabbit Alexa Fluor 594 (Life Technologies, Carlsbad, CA, USA), donkey anti-goat Alexa Fluor 488 (Life Technologies), or Streptavidin Alexa Fluor 555 (Life Technologies), at 1:500 dilutions. DAPI (4′,6-diamidine-2′-phenylindole dihydrochloride; Sigma-Aldrich) at 5 µg/mL was used to counterstain DNA and ensure nuclei visualization. Slides were mounted onto Vectashield (Vector Laboratories Inc., Burlingame, CA, USA), and images were acquired using a Laser Scanning Confocal Microscope Leica SP5II.

### 2.6. Histology Analysis

The length of the skin adipose tissue layer and the adipocyte diameter were determined using micrographs taken with a 4× magnification, “blindly”, under a light microscope Olympus CX31 coupled with a DP-25 Camera and Cell B software. For determination of the adipose layer length, two random regions of each image were measured from each of the three strains (*n* = 6). The adipocytes diameter was obtained by measuring 20 randomly chosen cells in three fields from three adipose tissue samples (*n* = 3 per group). All the analyses were performed using Fiji Software.

### 2.7. Scanning Electron Microscopy (SEM)

To assess tissue architecture, native and decellularized samples were fixed in 2.5% glutaraldehyde, in 0.1 M sodium cacodylate, for 30 min at RT, washed with cacodylate buffer, and dehydrated in a graded ethanol-water series. Carbon dioxide critical point drying was used to eliminate all humidity prior mounting each fragment onto sticky carbon tape. Samples were then coated with an Au/Pd thin film, by sputtering, using the SPI Module Sputter Coater equipment, and examined with a high-resolution scanning electron microscope with X-Ray Microanalysis JEOL JSM 6301F/Oxford INCA Energy 350.

### 2.8. Rheology Analysis

Viscoelastic properties of decellularized skin, kidney, and adipose tissue were assessed by rheological analysis using a Kinexus Pro rheometer (Malvern, Great Malvern, UK). All measurements were performed inside the equipment hood at 25 °C, using 4 mm upper and 8 mm sandblasted parallel plates. Linear viscoelastic regions (LVR) of samples were determined by performing frequency and amplitude strain sweeps. The complex shear modulus (G*) values were obtained at different frequency sweeps, with 0.03%, 0.39%, and 0.04% of shear strain for skin, kidney, and adipose tissue, respectively. Five animals of each strain, C57BL/6J, ob/ob, and db/db, were analyzed, with three to four matrices from each mouse being evaluated.

### 2.9. Atomic Force Microscopy (AFM)

Decellularized skin, kidney, adipose tissue, heart, and liver samples were fixed with formaldehyde before being embedded in optimal cutting temperature (OCT) medium, then frozen by means of liquid nitrogen, and kept at −80 °C. With the support of the Histology and Comparative Pathology Laboratory at Instituto de Medicina Molecular (iMM, Lisbon, Portugal), 30 µm-thick adjacent transversal slices were obtained. These tissue slices were used for indentation studies by AFM. All samples were mounted on super frost glass slides and stored at −80 °C until being analyzed on a NanoWizard 4 atomic force microscope (JPK Instruments, Berlin, Germany), mounted on top of an Axiovert 200 fluorescence inverted optical microscope (Zeiss, Jena, Germany). The elasticity differences throughout the decellularized matrices of each mice model (C57BL/6J, ob/ob, and db/db) were evaluated by using non-functionalized OMCL TR-400-type silicon nitride tips (Olympus, Tokyo, Japan), softest triangular cantilevers with a tip radius of approximately 15 nm, and a resonance frequency of 11 kHz. The thermal fluctuation method was used to calibrate the spring constant of the tips by assuming a value of 0.02 N/m. Before retraction, the maximum applied force was 2 nN. The elasticity for each distinct acellular tissue was measured using a 10 × 10-point grid (in a 100 × 100 μm square image) for a total of 100 points measured for each area and seven curves for each point. Of note, at least two different areas of the tissue were measured. Data collection for each force-distance cycle was performed at 3 Hz with a z-displacement range of 5 µm. All the experiments were performed at RT in phosphate buffered saline solution. Force spectroscopy data were analyzed to obtain the Young’s Modulus and the indentation depth of the different tissues from each mice model, using JPK Image Processing software v. 6.0.55 (JPK Instruments), by application of the Hertzian model.

### 2.10. Statistical Analysis

GraphPad Prism Software V8 was used to design all graphs and perform the statistical analysis. The non-parametric Kruskal–Wallis test was used to assess the statistical significance of mice weight and glycemia levels. The parametric Student’s *t*-test (paired *t*-test) or the one-way univariate analysis of variance (ANOVA) with the Bonferroni Correction test were employed to assess the significance of all other comparisons. Globally, a 5% significance was used.

## 3. Results

### 3.1. Mouse Monitoring Reveals That Only db/db Animals Display Sustained Hyperglycemia

**Figure 1:**
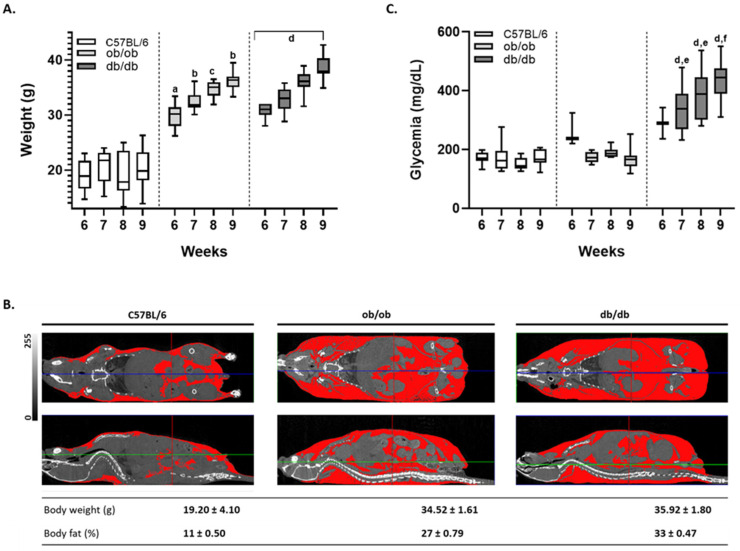
Only db/db animals presented sustained hyperglycemia despite both transgenic models developed the same levels of obesity. Follow up of the animal phenotype from 6 to 9 weeks of age. (**A**) Body weight comparison between C57BL/6J, ob/ob, and db/db animals, along time, maintained on a standard ad libitum diet. (**B**) Micron-scale computed tomography of 8-week-old C57BL/6J, ob/ob, and db/db animals. Representative images of coronal (upper panels) and sagittal (lower panels) orientations. Adipose tissue is highlighted in red, and the table depicts the percentage of body fat for the three animal strains. The grayscale indicates areas of density between 0 and 255 HU on the micro-CT slices shown. Coloring grids displayed are guides for anatomical comparison. (**C**) Time-dependent non-fasting blood glucose levels of C57BL/6J, ob/ob, and db/db models. Shown are the values of 18 age-matched control (white), ob/ob (light gray), and db/db (dark gray) animals, per time-point. Box-and-whisker plot represented by 25th and 75th quartiles, line within the box denotes the median, and the whiskers extend from minimum to maximum values. Statistical analyses of the weight and glycemia, across categories, were conducted using the median in the nonparametric Kruskal–Wallis test (a: C57BL/6J vs. ob/ob, *p* ≤ 0.0001; b: C57BL/6J vs. ob/ob, *p* ≤ 0.001; c: C57BL/6J vs. ob/ob, *p* < 0.05; d: C57BL/6J vs. db/db, *p* ≤ 0.0001; e: ob/ob vs. db/db, *p* ≤ 0.01; f: ob/ob vs. db/db, *p* ≤ 0.0001).

As a starting point to extensively characterize ob/ob and db/db mouse models, the weight and glycemia of all animals, from 6 to 9 weeks of age, was monitored. For control purposes, homozygous C57BL/6J animals from the respective transgenic litters were also analyzed. As expected, the weight of both ob/ob and db/db animals progressively increased with age, while that of their wild type counterparts remained almost unchanged (Figure 1A). The excessive weight of the genetically engineered strains was evident when evaluating in vivo microtomography images, which revealed a similar high degree of adipose tissue accumulation in both ob/ob and db/db animals (in red, Figure 1B). Moreover, the quantification of body fat indicated that the percentage of adipose tissue in ob/ob and db/db mice was comparable (27 ± 0.79% and 33 ± 0.47%), and substantially higher than that of background controls (11 ± 0.50%). Despite the obesity phenotype presented by both transgenic models, only the db/db animals displayed a pronounced and gradual glycemia increase (Figure 1C). Instead, ob/ob mice exhibited a non-significant raise of glycemia around 6 weeks of age, which returned to normal levels thereafter. These results highlight that db/db animals, presenting hyperglycemia, intimately mimic diabetes hallmarks, while ob/ob mice featured more an obesity model.

### 3.2. Histology Exposed Important Differences in Obese and Diabetic Tissues and Illustrated Decellularization Efficiency

**Figure 2:**
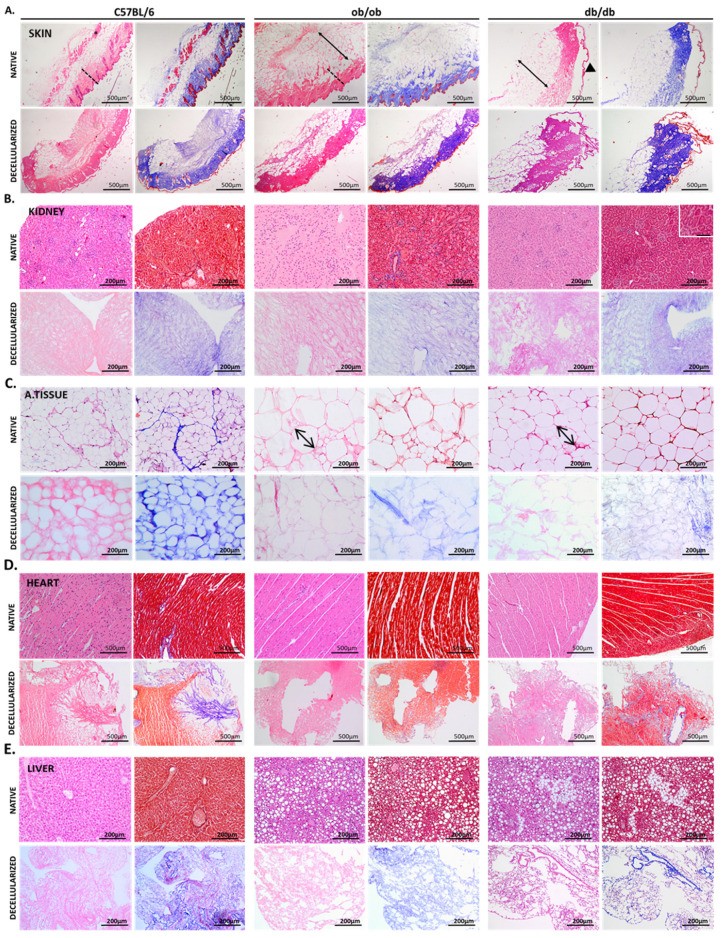
Native tissues of transgenic mice strains present evident histological alterations; however, ECM scaffolds are equally maintained after decellularization. Optical microscopy micrographs of native (upper panels) and decellularized (lower panels) tissue sections. Representative images of skin (**A**); kidney (**B**); adipose tissue (**C**); heart (**D**) and liver (**E**) samples obtained from C57BL/6J, ob/ob, and db/db animals. Depicted are histology H&E (left panels) and Masson’s Trichrome (right panels) stainings, with scale bars included in each image (200 or 500 µm as indicated, and 100 µm in the inset). Asterisk, skin muscle layer; thin arrow, skin adipose layer; dashed line, skin dermis; arrowhead, epidermis; bold arrow, adipocyte hypertrophy.

To compare the histological structures of ob/ob and db/db animals, paraffin embedded samples of skin, kidney, adipose tissue, liver, and heart, before and after decellularization, were sliced and stained with H&E and Masson’s Trichrome. As control, C57BL/6J animal organs and tissues were also processed for histological analysis. When analyzing the skin of the transgenic models, the most obvious difference was the expansion of the dermal adipose layer, with signs of adipocyte hyperplasia and hypertrophy (Figure 2A, arrow). In fact, ob/ob mice displayed the highest adipose layer size (7.12 ± 1.67 A.U.), when compared with db/db mice (5.50 ± 0.72 A.U.) or C57BL/6J (3.08 ± 0.56 A.U.) (Figure S1A,C). In addition, the muscle layer (asterisk) present in the skin of C57BL/6J mice seems to be vestigial in ob/ob, and totally absent in db/db animals. The dermis also appeared to be altered, possibly more compact (dashed line), in both obese and diabetic animals. Regarding the epidermis, the major alteration seems to occur in diabetic mice, where a loose architecture was observed (arrowhead). In decellularized skin matrices, the characteristics of the native tissues from each strain were maintained and the skin layers preserved. This indicates that the obtained scaffolds closely mimic native samples, regardless of their histological differences (Figure 2A). In addition, the DNA quantification clearly attested the effectiveness of the decellularization protocol, as almost no DNA was quantified in the matrices in comparison to the native skin samples (Figure S1D). Regarding the kidney histology, both ob/ob and db/db tissues exhibit signs of nephropathy (Figure 2B and Figure S1E) with extensive mesangial expansion (Figure S1E, asterisk) in both transgenic strains, due to increased ECM production, and diffuse thickening of the glomerular basement membrane in db/db animals (Figure S1E, arrow). Moreover, tubulointerstitial changes, corresponding to the thickening of tubular basement membranes and to the interstitial widening, were also observed, particularly in diabetic kidneys (Figure 2B, inset). In decellularized kidney samples, intact cells and cell debris were absent, as evidenced by H&E staining. Scaffolds displayed an exclusive blue staining in Masson’s Trichrome, indicating total loss of cellular constituents with collagen ECM preservation (Figure 2B). As previously mentioned, both transgenic animal strains suffered from a gradual weight increase, becoming obese at 5 weeks of age, although, only db/db presented sustained hyperglycemia. When analyzing the intraperitoneal adipose tissue of both obese and diabetic animals, adipocyte hypertrophy can be identified (Figure 2C, arrows), as supported by similar averages in adipocyte diameters in ob/ob and db/db (0.76 ± 0.011A.U. and 0.89 ± 0.18 A.U., respectively), when compared with wild type controls (0.38 ± 0.06 A.U.) (Figure S1B,C). This is a feature of a clear obese phenotype in both genetically engineered rodent strains. In decellularized matrices, adipocyte alterations were also evident, as their number and size in C57BL/6J animals were clearly distinct from those in transgenic animals (Figure 2C). Of note, adipose tissue of the three animal strains presented a gelatin-like texture and low cellularity, yet both the Masson’s Trichrome staining and the DNA quantification reveal that its ECM was successfully decellularized (Figure 2C and Figure S1D). In contrast, the analysis of animals’ heart tissue histology did not reveal major differences between the three murine strains (Figure 2D). As for the decellularized structures, the red staining in Masson’s Trichrome indicates that some heart muscle fibers were maintained, highlighting the density of the organ (Figure 2D). Yet, DNA presence in scaffolds was vestigial, confirming that this is indeed an acellular structure (Figure S1D). In the liver of both transgenic strains, evident steatosis (score 3 in the NAFLD Activity Score) with a low degree of hepatocyte ballooning and mild intralobular inflammation (Figure 2E) was observed. Upon decellularization, liver scaffolds retained their ECM fibrous proteins without an organized histological structure. Nevertheless, both ob/ob and db/db matrices appeared to be looser than those of their wild type controls (Figure 2E). Altogether, the data extracted provide a detailed histopathological characterization of ob/ob and db/db mice models, illustrating major differences in organs and tissues such as skin, kidney, adipose tissue, heart, and liver from obese and diabetic animals. Interestingly, db/db organs were those that revealed the most prominent structural alterations, suggesting that diabetic factors are more associated with pathology worsening than obesity.

### 3.3. Scanning-Electron Microscopy Revealed Native and Decellularized Mouse Tissues Architecture Features

**Figure 3:**
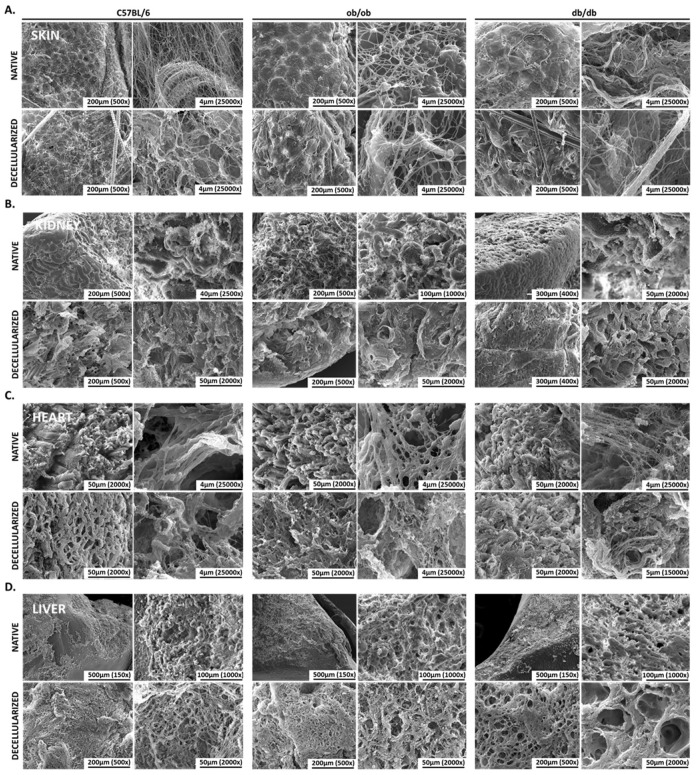
Decellularized mice tissues maintain their native architecture. Scanning electron microscopy images of native (upper panels) and decellularized (lower panels) mice tissue sections. Representative micrographs of skin (**A**); kidney (**B**); heart (**C**), and liver (**D**) from C57BL/6J, ob/ob, and db/db animals. Scale bars are indicated in the micrographs (magnification ranging from 150× to 25,000×, spatial resolution of 4 to 500 µm).

When characterizing tissues and extracellular matrices, architecture is often dismissed. Nonetheless, it is a relevant feature complementary to histological analysis. Thus, scanning electron microscopy was performed to understand whether native and decellularized tissues from wild type, ob/ob, and db/db animals exhibit ultrastructural differences. The architecture of the native skin revealed that keratinocytes are organized in a honeycomb-like structure visible in the three mice strains. Yet, in obese, and particularly in diabetic animals, keratinocytes are larger in size and smaller in number, when compared with wild type controls (Figure 3). Additionally, in db/db mice the skin structure seems to be less compact and more disorganized than in the other models, in line with histology data. The fibers that constitute the skin can be observed in more detail at the surface of the tissue. It is clear that, in C57BL/6J mice, fibers are thinner than in the transgenic strains. Upon decellularization, the main architecture is kept, but these fibers are slightly more aggregated. Still, the overall morphology of the skin ECM resembles that of native conditions (Figure 3A). The kidney tubular architecture was evident in the three animal strains, both in native and in decellularized tissues. The glomeruli are noticeable structures wrapped in the renal tubes observed in the native tissue but not in decellularized kidney samples. The meshwork structure of the kidney composed of strands and pores was evident in acellular tissues, indicating that kidney main structural elements were preserved after decellularization (Figure 3B). Native heart surface analysis revealed that the parallel fibers of the contractile muscle were readily detected and similar in the three animal models. Nevertheless, at higher magnifications, the fibrous architecture appeared thicker in wild type than in obese and diabetic mice, indicating mild structural differences in transgenic strains. In heart-decellularized matrices, the fibrous architecture was maintained, but slightly more disorganized, with noticeable holes resulting from nuclei rupture. No major differences were observed among control C57BL/6J, ob/ob, and db/db heart samples (Figure 3C). In the liver, a marked steatotic parenchyma, in both obese and diabetic mice, was observed (Figure 3D). The fat pockets previously identified in native tissues are illustrated by holes in the liver of both ob/ob and db/db mice. This cavernous-like surface was more evident upon decellularization. In transgenic models, nuclei absence together with the holes left from the fat depots, created an organized pore-containing matrix. Strikingly, db/db liver ECM presented larger pores than that of ob/ob matrices, suggesting that the liver parenchyma of these animals accumulates higher levels of fat. It should be noticed that, due to the liquid-like nature of the intraperitoneal adipose tissue, it was not possible, in our conditions, to obtain reliable SEM data for adipose tissue samples of the three mice strains. Overall, the analysis of tissue architecture pinpointed interesting differences in obese and diabetic tissues and, more importantly, confirmed that decellularization does not alter the main ECM structure of C57BL/6J, ob/ob, and db/db skin, kidney, heart, and liver.

### 3.4. Decellularized Matrices from ob/ob and db/db Animals Retained Major ECM Components

**Figure 4:**
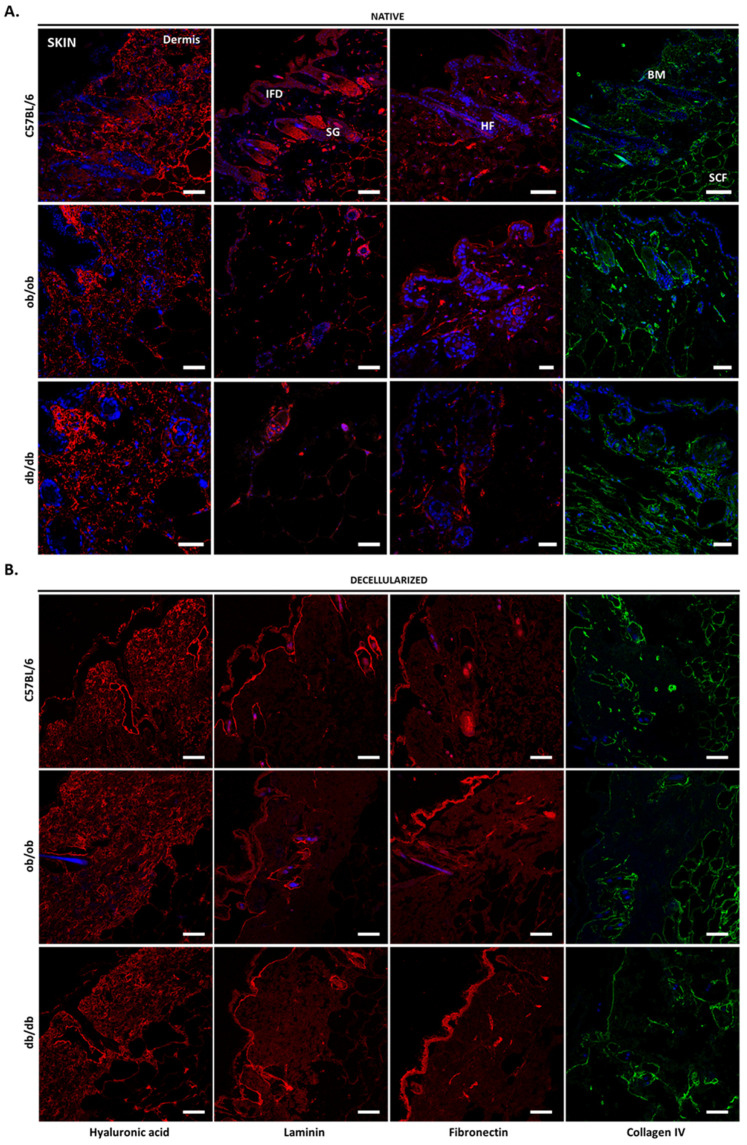
ECM components of the skin before and after decellularization. Visualization of native (**A**) and decellularized (**B**) skin sections from C57BL/6J, ob/ob, and db/db mice. The representative results compare confocal microscopy images of hyaluronic acid (first column), laminin (second columns), fibronectin (third column), and collagen IV (forth column) immunostained skin samples. Nuclei are shown in blue after DAPI counterstaining. Mouse hair may also be observed in blue as it emits light in the blue spectral range. IFD, interfollicular dermis; SG, sebaceous gland; HF, hair follicle; BM, basement membrane; SCF, subcutaneous fat layer. Scale bar: 50 μm.

To further characterize the native and decellularized tissues, we examined, by immunofluorescence, the composition, and distribution of four major ECM components: hyaluronic acid, laminin, fibronectin, and collagen type IV. In the skin of C57BL/6J, ob/ob, and db/db animals, hyaluronic acid was present at the basement membrane, though mainly in the papillary and reticular dermis, as well as the subcutaneous fat layer, where it surrounds adipocytes. This distribution was maintained in the decellularized matrices (Figure 4). Laminin, which presented decreased expression in obese non-diabetic and diabetic mice (Figure 4A), located at the hair follicles, where it is known to regulate the dermal papilla function. The hair follicle localization of laminin is especially noticeable in the decellularized skin (Figure 4B). This ECM protein was also found in sebaceous glands and, to a less extent, in the interfollicular epidermis. Fibronectin presented a scattered pattern throughout all skin layers, with no noticeable differences among strains, either in native or decellularized tissues. As for collagen IV, it was observed to be the major constituent of the basement membrane, hair follicles, and subcutaneous fat layer (Figure 4A) [16].

**Figure 5:**
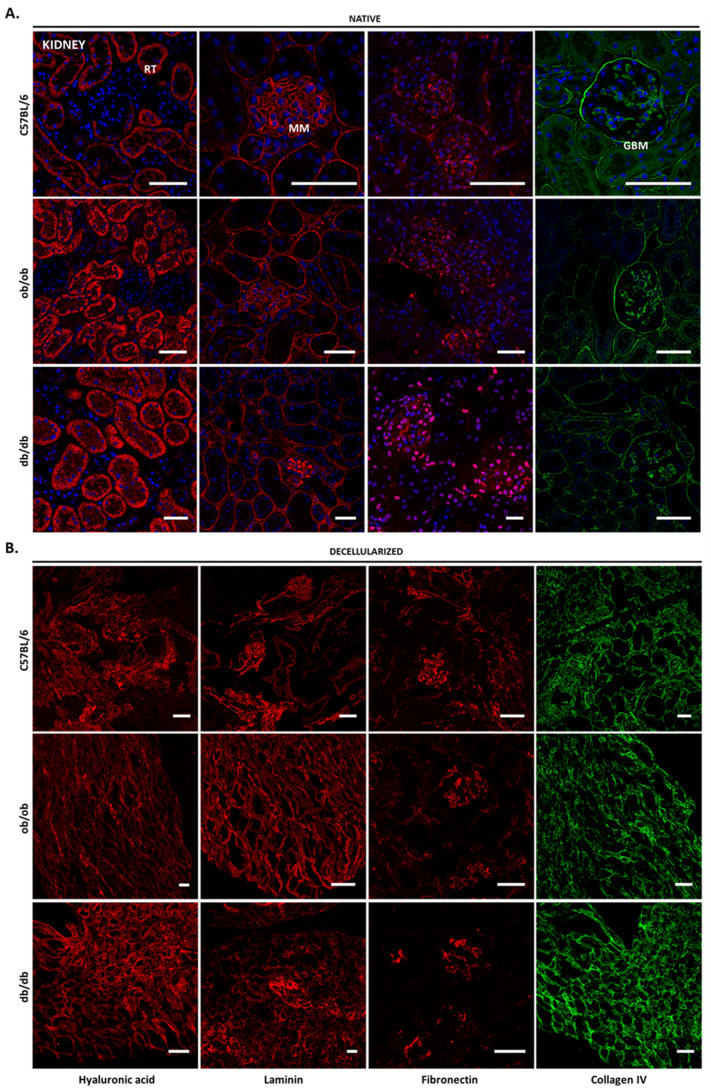
Kidney ECM components prior to and after decellularization. Representative confocal microscopy images of native (**A**) and decellularized (**B**) kidney sections from C57BL/6J, ob/ob, and db/db mice. Confocal microscopy images of immunostaining for hyaluronic acid (first column), laminin (second column), fibronectin (third column), and collagen IV (forth column). Nuclei are stained in blue (with DAPI). RT, renal tubules; MM, mesangial matrix; GBM, glomerular basement membrane. Scale bar: 100 μm.

In the kidney, the most striking difference was the accumulation of laminin in the mesangial matrix of db/db animals. Apart from laminin, the mesangial ECM is composed of collagen type IV and fibronectin (Figure 5A). The glomerular basement membrane is constituted by laminin and collagen IV that entangle to maintain the integrity of the structure. The presence of hyaluronic acid is mainly restricted to renal tubules and appears to be thicker in diabetic mice. Upon decellularization, the obtained scaffolds maintained all ECM components studied (Figure 5B); however, their physiological location was difficult to identify, with the exception of fibronectin that was clearly kept within the mesangial matrix.

**Figure 6:**
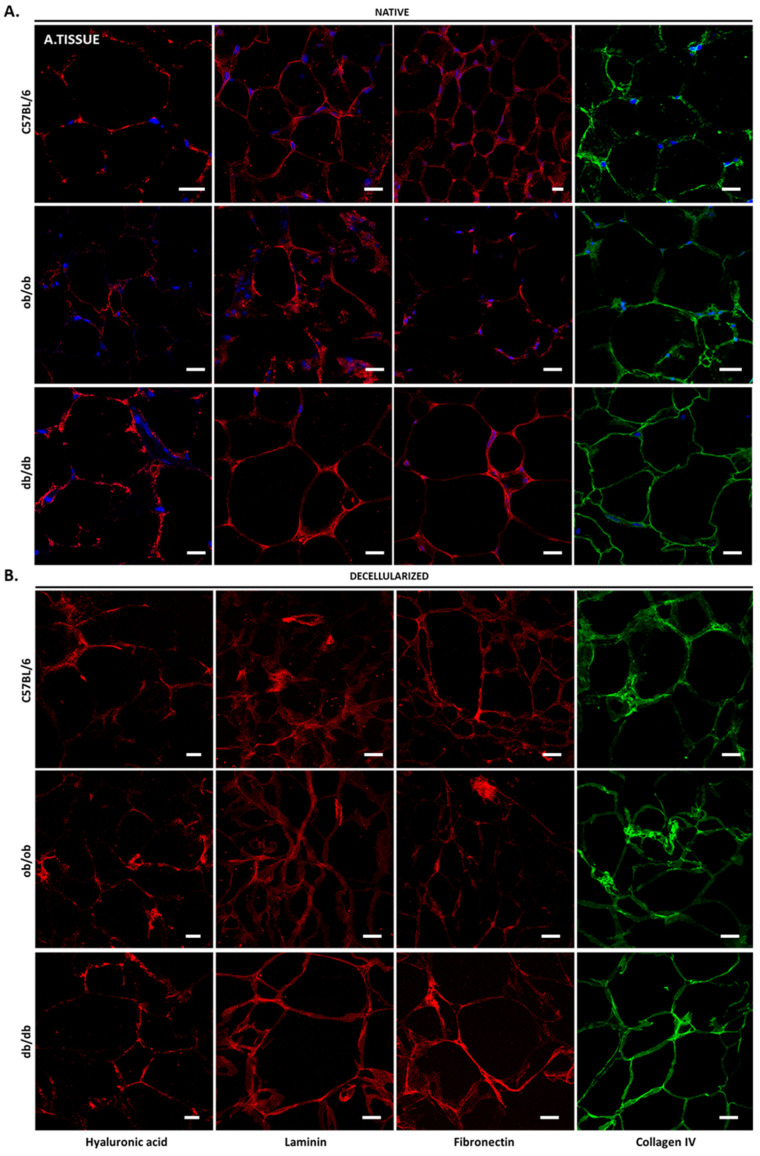
Acellular intraperitoneal adipose tissue retains its ECM components. Confocal microscopy images of native (**A**) and decellularized (**B**) adipose tissue sections from C57BL/6J, ob/ob, and db/db mice. Shown are immunofluorescence results for hyaluronic acid (first column), laminin (second column), fibronectin (third column), and collagen IV (forth column). DAPI staining indicates cell nuclei. Scale bar: 100 μm.

Concerning the adipose tissue, the ECM surrounding each adipocyte, designated as basal lamina, contained hyaluronic acid, laminin, fibronectin, and collagen type IV (Figure 6), although differently distributed in C57BL/6J, ob/ob, and db/db animals. While in native samples wild type mice presented less collagen IV than transgenic animals, in obese non-diabetic individuals, a reduction of hyaluronic acid and fibronectin was observed in comparison with C57BL/6J controls and db/db mice. This suggests the existence of clear differences among the intraperitoneal fat depots of each animal strain. In acellular adipose tissue, the presence of the four matrices components analyzed was maintained, with the native cues being kept in each scaffold (Figure 6B).

**Figure 7:**
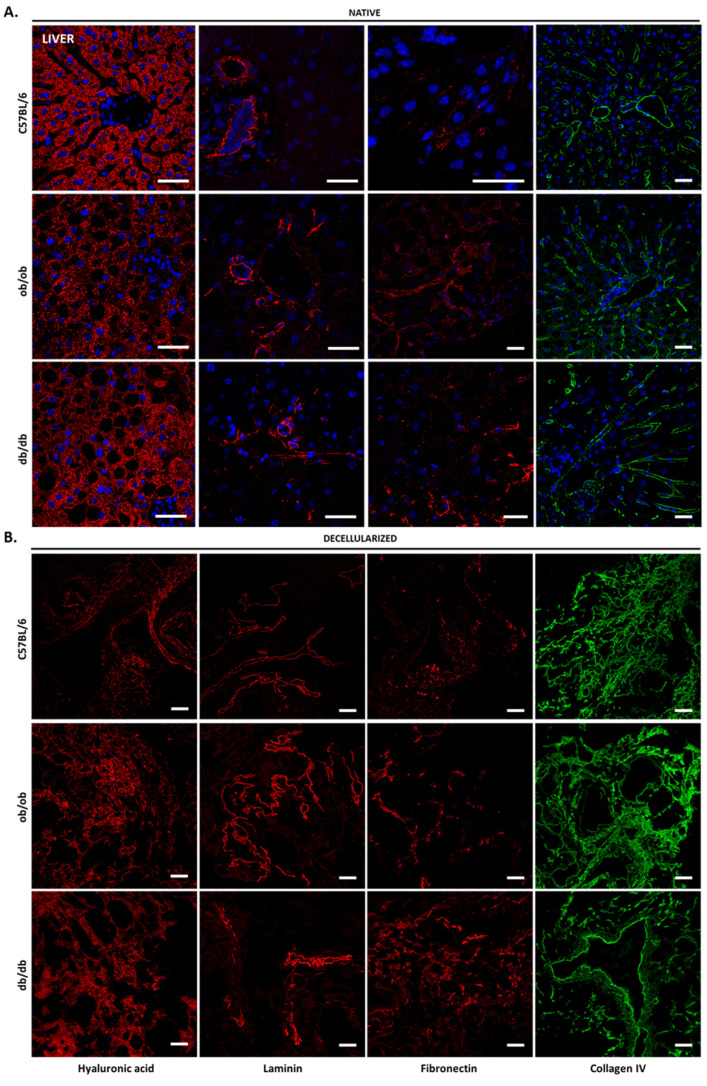
ECM components of native and decellularized liver. Immunohistochemical staining of ECM components from native (**A**) and decellularized (**B**) liver sections of C57BL/6J, ob/ob, and db/db mice. Confocal microscopy images of hyaluronic acid (first column), laminin (second column), fibronectin (third column), and collagen IV (forth column) immunostained liver samples. Nuclei are stained in blue (with DAPI). Scale bar: 100 μm.

The evaluation of heart ECM traits revealed that hyaluronic acid and laminin are evenly distributed throughout the myocardium, while fibronectin exhibited a lower and heterogeneous expression restricted to the vicinity of endothelial cells (Figure S2). Collagen type IV surrounded the cardiomyocytes that compose the native tissue and the vessels found in C57BL/6J, ob/ob, and db/db samples. Heart scaffolds presented an abundant hyaluronic acid and collagen IV content but had a mild presence of laminin and fibronectin. Nevertheless, the ECM component profile in heart matrices resembled that of native tissues (Figure S2B). Finally, the staining of the ECM components in native liver sections exposed the location of each constituent (Figure 7). Hyaluronic acid was clearly restricted to the hepatocyte cytoplasm, even in obese and diabetic steatotic parenchyma. Laminin displayed an expression restricted to the interlobular space and interlobular veins, while fibronectin seemed to surround the cells that locate at the sinusoids. Collagen type IV was expressed in the sinusoid compartment and around vessel walls. The increased levels of collagen type IV present in the liver were easily observed in decellularized matrices (Figure 7B). The latter retained the ECM molecules composing the native tissue, albeit with a more diffuse distribution.

Of note, the absence of tissue auto-fluorescence and secondary antibody non-specific binding (Figure S3) indicated that each ECM component was specifically detected. Collectively, the results gathered here show that decellularized matrices from all animal strains preserve the native tissue ECM components and resemble the original morphology. Importantly, although protein location is maintained upon decellularization in obese and diabetic tissues, the levels of each matrix constituent may be altered when compared to C57BL/6J controls. Exemplifying this is the evident decrease of laminin in the skin of ob/ob and db/db animals versus wild type, the laminin accumulation in db/db kidney structures, and the apparent decrease of hyaluronic acid in hepatocyte cytoplasm of both obese and diabetic mice.

### 3.5. ECM from Obese and Diabetic Animals Exhibit Distinct Viscoelastic Properties

**Figure 8:**
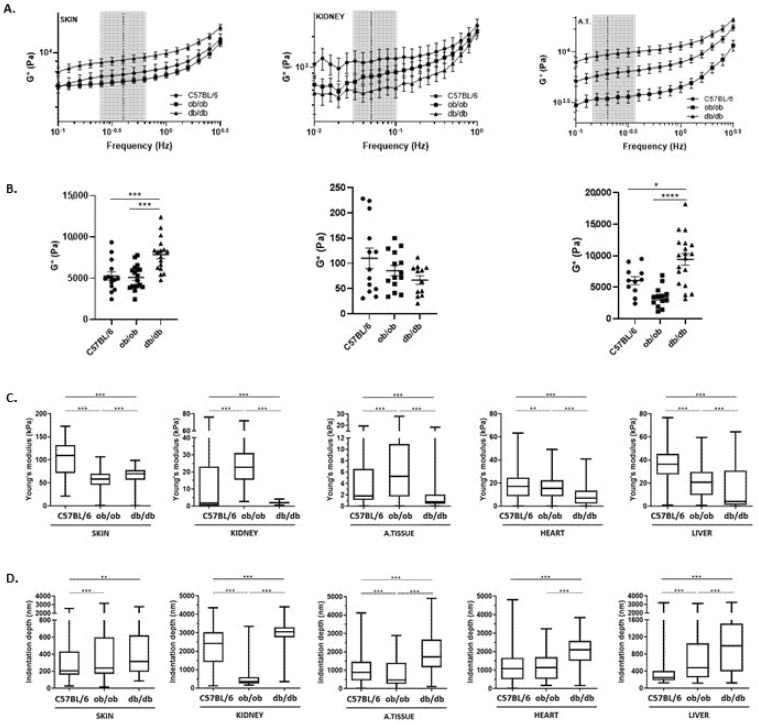
Obese and diabetic mouse tissues present distinct viscoelastic properties. (**A**) Complex shear modulus amplitude (G*) values of the skin, kidney, and adipose tissue matrices obtained at different frequency sweeps, with 0.03%, 0.39%, and 0.04% of shear strain, respectively. Represented are the results from C57BL/6J (circles), ob/ob (squares), and db/db (triangles) animal strains. (**B**) Complex shear modulus of skin, kidney, and adipose tissue scaffolds assessed from the linear viscoelastic region (LVR, gray area) of the performed frequency sweeps (dotted line) represented in a scatter dot plot. Each reading is the average from five animals: C57BL/6J (circles), ob/ob (squares), and db/db (triangles) strains (3 to 4 matrices each) ± SEM. Statistical analyses were assessed using one-way ANOVA * *p* < 0.05; *** *p* ≤ 0.001; **** *p* ≤ 0.0001. (**C**) Atomic force microscopy of skin, kidney, fat, heart, and liver matrices from C57BL/6J, ob/ob, and db/db mice. Represented is the Young’s modulus and (**D**) indentation depth extracted from acquired AFM force indentation curves, after applying the Hertzian model. Statistical evaluation was performed using one-way ANOVA with the Bonferroni Correction Test, ** *p* ≤ 0.001 and *** *p* ≤ 0.0001.

To evaluate the effect of diabetes on the ECM viscoelastic properties, skin, kidney, and adipose tissue matrices from C57BL/6J, ob/ob, and db/db animals were analyzed by rheology. First, we determined the linear viscoelastic region (LVR) by performing both frequency and amplitude sweeps on three independent samples from skin, kidney, and adipose tissue matrices. Such evaluation revealed that frequency sweeps ranged from 0.01 Hz to 3 Hz, and the shear strain was 0.03%, 0.39%, and 0.04% for skin, kidney, and adipose tissue, respectively (Figure 8). In addition, we observed that the viscous component (G″, imaginary part) is smaller than the elastic component (G′, real part) of the complex shear modulus in approximately one order of magnitude in the three tissues analyzed (Figure S4). Secondly, the amplitude of the complex shear modulus (G*) was determined along each frequency range and, from the LVR, the value of each matrix G* was retrieved and compared among mice strains (Figure 8A,B). Interestingly, the skin ECM G* amplitude was significantly higher in diabetic animals than in obese or wild type mice. This may relate with the alterations found through histology, i.e., we expect that the muscle layer loss together with the compact dermis, observed in the skin of db/db murine model, results in the increase of ECM rigidity (Figure 8B). The comparison between kidney matrices revealed no statistically significant differences. Yet, there was a tendency for lower G* on the db/db ECM, opposed to a higher complex shear modulus amplitude on the C57BL/6J matrices (Figure 8B). Importantly, when analyzing the intraperitoneal adipose tissue of each animal strain, striking differences were found between obese and diabetic mice. The adipose depots in db/db animals presented higher G* amplitude than those in ob/ob, although both strains present the same weight levels and fat accumulation (Figure 1). To our best knowledge, this data demonstrates for the first time that obese and diabetic intraperitoneal adipose tissue display important biomechanical differences regarding their ECM. Thus, the morphological alterations observed in ob/ob and db/db matrices are sufficiently dramatic to produce biomechanical alterations, as observed above [17,18,19]. However, to further explore the differences on the mechanical properties of scaffolds at a smaller scale, decellularized matrices from each organ and tissue were evaluated by atomic force microscopy (AFM). For that, formaldehyde-fixed samples were embedded in OCT and transversal slices with 30 µm were analyzed for their Young’s Modulus values (stiffness parameter), as well as indentation depth (Figure 8C,D, respectively). Interestingly, when comparing the Young’s Modulus values of C57BL/6J, obese, and diabetic mice samples, statistically significant differences were identified in all matrices. The db/db acellular tissues, with the exception of the skin, presented the lowest value of Young’s Modulus, indicating that diabetic ECM presents the lowest stiffness (highest tissue elasticity) when compared with obese or wild type controls (Figure 8C). Obese kidney and adipose tissue ECM displayed higher stiffness than the same tissues of diabetic and C57BL/6J mice. These results point out, once again, relevant differences between the ob/ob and db/db animal models. As for the skin, AFM measurements were restricted to the epidermis as this was the region with the most profound histological alterations. Collected data indicated that C57BL/6J matrices were those with the highest Young’s Modulus, contrary to rheometry measurements. However, the stiffness of the skin determined by AFM was significantly higher in diabetic mice than in obese mice, as shown before in rheology studies by the G* amplitude values. As for the indentation depth (Figure 8D), the values obtained are in line with those of the Young’s Modulus: higher indentation depth associates with lower Young’s Modulus values (i.e., higher elasticity of the sample). In summary, the data obtained herein on the mechanical properties of the evaluated matrices strongly highlight substantial differences between C57BL/6J, obese, and diabetic ECMs. Knowing that biomechanical features have an important role in cell organization, profile, and function, it is thus essential to further investigate the biological impact of these biomechanical contrasts.

## 4. Discussion

In the current study, we have extensively characterized the diabetic db/db and the obese ob/ob animal models, detailing the histological and biomechanical features of the skin, kidney, liver, heart, and adipose tissue ECMs. Our analysis not only evidenced significant differences in the two transgenic mice strains in comparison with C57BL/6J controls, but also, and more importantly, it disclosed striking modifications specific of diabetic organs and tissues. Such data highlights the critical role of diabetes-associated features (e.g., sustained hyperglycemia) in ECM remodeling and points to a straight association of ECM properties with T2DM pathophysiology. The ob/ob (LeptinR^mut^) and db/db (Leptin^mut^) mice strains have mutations in leptin signaling resulting in uncontrolled satiety and reduced energy expenditure [20,21]. As described in the literature, and confirmed by us, such abnormalities render these rodents into an acute and early-onset obese condition [22]. Accordingly, we observed that intraperitoneal adipose tissue of both transgenic strains exhibited increased adipocyte size. Moreover, ob/ob and db/db liver examination revealed histological alterations similar to those of nonalcoholic fatty liver disease, a pathology associated with overt obesity and also present in diabetes [23,24]. Although both genetically engineered murine strains develop similar obese features, only db/db animals exhibit diabetes features, such as severe hyperglycemia. The levels of circulating glucose are known to be associated with alterations in ECM components leading, for example, to protein glycation. Subsequently, such modifications may result in changes of biomechanical properties of the tissue matrices [2,3,5], which in turn may modulate cell function. Nevertheless, the effect of diabetes on tissue matrices biomechanical features has mainly been assessed in native tissues [10,11,12]. Our data, using both native and decellularized samples, pinpointed important structural and biomechanical alterations in the diabetic skin, kidney, adipose tissue, heart, and liver. The epidermis of db/db displayed a loose structure that occurs, most likely, due to the irregular basal layer alignment, a feature already observed in streptozotocin-induced diabetic mouse models [25]. In addition, SEM analysis revealed that diabetic mice skin has a less compact architecture with decreased keratinocyte numbers than that of wild type mice, as previously reported [25]. The dermis and adipose layer of the db/db skin also hold clear structural differences when compared with C57BL/6J tissues. However, changes might be associated with overt obesity, as they were observed in both transgenic rodent models. In fact, it was already reported that the thickness of the different dermis layers’ changes when animals are subjected to a high-fat diet [26]. Apart from the skin, db/db kidneys also exhibit clear signs of diabetic renal disease, particularly in glomeruli and tubulointerstitial structures, abnormalities ascribed to diabetic nephropathy [27,28]. Of note, although in ob/ob the lack of hyperglycemia has been associated with the absence of diabetic nephropathy [22], we detected mild signs of kidney mesangial expansion, suggesting a degree of renal illness in this model. Another common complication of diabetes is the development of liver chronic disease, characterized by excessive accumulation of hepatic fat [23,24], as patent on the db/db steatotic liver parenchyma. The diabetic hepatic sinusoids, unlike other tissues, do not display typical fibrotic features, such as basement membrane thickening [29]. This is corroborated by our results showing no evident alterations in the levels of ECM components among wild type, obese, and diabetic tissues. As mentioned above, both transgenic mouse models feature overt obesity. Therefore, we performed a detailed analysis of intraperitoneal adipose tissue that revealed evident differences between the fat depots of db/db and ob/ob in comparison with C57BL/6J. Our data highlight adipocyte hypertrophy and higher expression of collagen type IV in obese and diabetic tissues, as well as decreased levels of hyaluronic acid restricted to ob/ob fat depots [30,31]. Of note, we were unable to address the architecture of intraperitoneal adipose tissue scaffolds by SEM [31]. When studying the heart, it was expected to detect signs of diabetes-associated cardiomyopathy in diabetic animals [2]. Yet, in our experimental setup, no major differences were found in the heart structure or in its ECM components. This probably occurs because the profibrotic signaling cascades that lead to myocardial fibrosis are activated later in life. In fact, the molecular mechanisms associated with diabetic cardiomyopathy were described only in 16-week-old animals, a stage where the disease is already chronic [2], instead of the 8-week-old mice used in this study. Of note, one should not exclude that obese and diabetic tissue histological differences selectively impact the decellularization efficiency. Nevertheless, our meticulous characterization of skin, kidney, intraperitoneal adipose tissue, heart, and liver decellularized matrices from wild type, obese, and diabetic animals strongly suggests that the main ECM structure, architecture, and composition present in native tissues is maintained in decellularized scaffolds. Therefore, we took advantage of such matrices and evaluated the impact of diabetes condition on ECM biomechanical properties. Our results unveiled significant differences among the complex shear modulus, Young’s Modulus, elasticity, and rigidity of wild type, obese, and diabetic acellular tissues. The diabetic intraperitoneal adipose tissue scaffolds presented the highest complex shear modulus amplitude and the lowest Young’s Modulus when compared with obese or wild type controls. To our knowledge, this is the first time that the diabetic intraperitoneal adipose tissue ECM is shown to display critical biomechanical differences. Importantly, db/db kidney, heart, and liver matrices exhibited the lowest Young’s Modulus values, showing that diabetic scaffolds possess decreased stiffness and high tissue elasticity than obese or wild type matrices. Interestingly, the changes in the stiffness of heart ECM are the opposite of what was reported by Michaelson et al. when analyzing the native diastolic stiffness, diastolic left ventricular rigidity, and myocyte Young’s Modulus [12], underlining the relevance of evaluating the viscoelastic properties of the decellularized tissue. The evaluation of the skin biomechanical properties by both rheology and AFM revealed conflicting results. The diabetic ECM showed high complex shear modulus but also low Young’s Modulus when compared with both obese and, particularly, wild type samples. These contradictory data on the skin stiffness might be explained by the fact that in rheology all skin layers, namely epidermis, dermis, and hypodermis, were analyzed as a whole, while in AFM only the epidermis layer was considered. Moreover, there are intrinsic differences between the employed techniques (rheology vs. AFM). Rheology relies on the deformability of the whole skin matrices at a given shear stress, while AFM is dependent on a sharp probe tip (or cantilever) that scans over a surface and senses the viscoelastic shear forces at the nanometer scale. As such, the impact of the skin complex histology and the areas of the skin tissue where the AFM probing was performed could be highly relevant for the AFM data obtained, resulting in a stiffness readout different from that of rheometry. Moreover, the fact that all tissues for AFM analyses were fixed in formaldehyde, prior to embedment in OCT, might have affected tissue stiffness. Nevertheless, one may argue that our rheology results are in line with the increased stiffness encountered in the native plantar skin from diabetic patients in comparison with that of non-diabetic individuals [10]. Importantly, the biomechanical studies clearly show that the most noticeable differences in the analyzed matrices were detected in diabetic tissues, suggesting that T2DM-associated features promote impactful ECM alterations. In summary, diabetic and obese animal models present significant differences in their skin, kidney, intraperitoneal adipose tissue, heart, and liver morphology, and composition. Moreover, rheology and AFM analysis of the referred tissues ECM revealed important biomechanical differences, being more pronounced in the diabetic animals, which exhibit significantly different stiffness and viscoelastic properties when compared with normal (C57BL/6J) or obese (ob/ob) matrices. Such results reinforce the fact that changes in ECM structure and biomechanical properties may be involved in the pathophysiological process of diabetic complications [4,9,10,13].

## Data Availability

Not applicable.

## Supplementary Materials

The following are available online at https://www.mdpi.com/article/10.3390/biomedicines10010057/s1, Figure S1: Histology measurements and DNA quantification of decellularization scaffolds, Figure S2: Heart ECM components before and after decellularization, Figure S3: Negative stainings of parafin-embedded tissues, Figure S4: Elastic and viscous components of each matrix.

## References

[1] Khawandanah J. (2019). Double or hybrid diabetes: A systematic review on disease prevalence, characteristics and risk factors. Nutr. Diabetes.

[2] Zhao J., Randive R., Stewart J.A. (2014). Molecular mechanisms of AGE/RAGE-mediated fibrosis in the diabetic heart. World J. Diabetes.

[3] Brownlee M. (1991). Glycosylation products as toxic mediators of diabetic complications. Annu. Rev. Med..

[4] Chawla D., Bansal S., Banerjee B.D., Madhu S.V., Kalra O.P., Tripathi A.K. (2014). Role of advanced glycation end product (AGE)-induced receptor (RAGE) expression in diabetic vascular complications. Microvasc. Res..

[5] Rojas A., Añazco C., González I., Araya P. (2018). Extracellular matrix glycation and receptor for advanced glycation end-products activation: A missing piece in the puzzle of the association between diabetes and cancer. Carcinogenesis.

[6] Bansode S.B., Gacche R.N. (2019). Glycation-induced modification of tissue-specific ECM proteins: A pathophysiological mechanism in degenerative diseases. Biochim. Biophys. Acta Gen. Subj..

[7] Rodrigues L., Matafome P., Crisóstomo J., Santos-Silva D., Sena C., Pereira P., Seiça R. (2014). Advanced glycation end products and diabetic nephropathy: A comparative study using diabetic and normal rats with methylglyoxal-induced glycation. J. Physiol. Biochem..

[8] Kuhad A., Singh P., Chopra K. (2015). Matrix metalloproteinases: Potential therapeutic target for diabetic neuropathic pain. Expert Opin. Ther. Targets.

[9] Ban C.R., Twigg S.M. (2008). Fibrosis in diabetes 676 complications: Pathogenic mechanisms and circulating and urinary markers. Vasc. Health Risk Manag..

[10] Pai S., Ledoux W.R. (2010). The compressive mechanical properties of diabetic and non-diabetic plantar soft tissue. J. Biomech..

[11] Boivin G.P., Elenes E.Y., Schultze A.K., Chodavarapu H., Hunter S.A., Elased K.M. (2014). Biomechanical properties and histology of db/db diabetic mouse Achilles tendon. Muscles Ligaments Tendons J..

[12] Michaelson J., Hariharan V., Huang H. (2014). Hyperglycemic and hyperlipidemic conditions alter cardiac cell biomechanical properties. Biophys. J..

[13] Van Heerebeek L., Hamdani N., Handoko M.L., Falcao-Pires I., Musters R.J., Kupreishvili K., Ijsselmuiden A.J.J., Schalkwijk C.G., Bronzwaer J.G.F., Diamant M. (2008). Diastolic stiffness of the failing diabetic heart: Importance of fibrosis, advanced glycation end products, and myocyte resting tension. Circulation.

[14] Pinto M.L., Rios E., Silva A.C., Neves S.C., Caires H.R., Pinto A.T., Duraes C., Carvalho F.A., Cardoso A.P., Santos N.C. (2017). Decellularized human colorectal cancer matrices polarize macrophages towards an anti-inflammatory phenotype promoting cancer cell invasion via CCL18. Biomaterials.

[15] Silva A.C., Rodrigues S.C., Caldeira J., Nunes A.M., Sampaio-Pinto V., Resende T.P., Oliveira M.J., Barbosa M.A., Thorsteinsdóttir S., Nascimento D.S. (2016). Three-dimensional scaffolds of fetal decellularized hearts exhibit enhanced potential to support cardiac cells in comparison to the adult. Biomaterials.

[16] Watt F.M., Fujiwara H. (2011). Cell-extracellular matrix interactions in normal and diseased skin. Cold Spring Harb. Perspect. Biol..

[17] Travasso R.D., Buxton G.A., Kuksenok O., Good K., Balazs A.C. (2005). Modeling the morphology and mechanical properties of sheared ternary mixtures. J. Chem. Phys..

[18] Arevalo R.C., Urbach J.S., Blair D.L. (2010). Size-dependent rheology of type-I collagen networks. Biophys. J..

[19] Juliar B.A., Strieder-Barboza C., Karmakar M., Flesher C.G., Baker N.A., Varban O.A., Lumeng C.N., Putnam A.J., O’Rourke R.W. (2020). Viscoelastic characterization of diabetic and non-diabetic human adipose tissue. Biorheology.

[20] Drel V.R., Mashtalir N., Ilnytska O., Shin J., Li F., Lyzogubov V.V., Obrosova I.G. (2006). The leptin deficient (ob/ob) mouse: A new animal model of peripheral neuropathy of type 2 diabetes and obesity. Diabetes.

[21] Burke S.J., Batdorf H.M., Burk D.H., Noland R.C., Eder A.E., Boulos M.S., Karlstad M.D., Collier J.J. (2017). db/db mice exhibit features of human type 2 diabetes that are not present in weight-matched C57BL/6J mice fed a western diet. J. Diabetes Res..

[22] Wang B., Chandrasekera P.C., Pippin J.J. (2014). Leptin- and leptin receptor-deficient rodent models: Relevance for human type 2 diabetes. Curr. Diabetes Rev..

[23] Kleiner D.E., Brunt E.M., Van Natta M., Behling C., Contos M.J., Cummings O.W., Ferrel L.D., Liu Y.C., Torbenson M.S., Unalp-Arida A. (2005). Design and validation of a histological scoring system for nonalcoholic fatty liver disease. Hepatology.

[24] Rath M.M., Panigrahi M.K., Pattnaik K., Bhuyan P., Kar S.K., Misra B., Misra D., Meher C., Agrawal O., Rath J. (2016). Histological evaluation of non-alcoholic fatty liver disease and its correlation with different noninvasive scoring systems with special reference to fibrosis: A single center experience. J. Clin. Exp. Hepatol..

[25] Okano J., Kojima H., Katagi M., Nakagawa T., Nakae Y., Terashima T., Kurakane T., Kubota M., Maegawa H., Udagawa J. (2016). Hyperglycemia induces skin barrier dysfunctions with impairment of epidermal integrity in non-wounded skin of type 1 diabetic mice. PLoS ONE.

[26] Nguyen-Tu M.S., Nivoit P., Oréa V., Lemoine S., Acquaviva C., Pagnon-Minot A., Fromy B., Sethi J.K., Siguado-Roussel D. (2019). Inflammation-linked adaptations in dermal microvascular reactivity accompany the development of obesity and type 2 diabetes. Int. J. Obes..

[27] Ha T.S., Barnes J.L., Stewart J.L., Ko C.W., Miner J.H., Abrahamson D.R., Sanes J.R., Kasinath B.S. (1999). Regulation of renal laminin in mice with type II diabetes. J. Am. Soc. Nephrol..

[28] Liu Y., Wang Z., Yin W., Li Q., Cai M., Zhang C., Xiao J., Hou H., Li H., Zu X. (2007). Severe insulin resistance and moderate glomerulosclerosis in a minipig model induced by high-fat/ high-sucrose/ high Cholesterol diet. Exp. Anim..

[29] Jović M., Nikolić I., Todorović V., Petrović A., Petrović V., Mojsilović M., Denčić T. (2018). Distribution of collagen I, III, and IV and laminin in the human liver during prenatal development. Cells Tissues Organs.

[30] Mariman E.C., Wang P. (2010). Adipocyte extracellular matrix composition, dynamics and role in obesity. Cell Mol. Life Sci..

[31] Song M., Liu Y., Hui L. (2018). Preparation and characterization of acellular adipose tissue matrix using a combination of physical and chemical treatments. Mol. Med. Rep..

