# Supplementary figures and images for: Mice with Type 2 Diabetes Present Significant Alterations in Their Tissue Biomechanical Properties and Histological Features
Source: Biomedicines. 2021 Dec 28;10(1):57. doi: 10.3390/biomedicines10010057 (PMC8773308; doi:10.3390/biomedicines10010057)

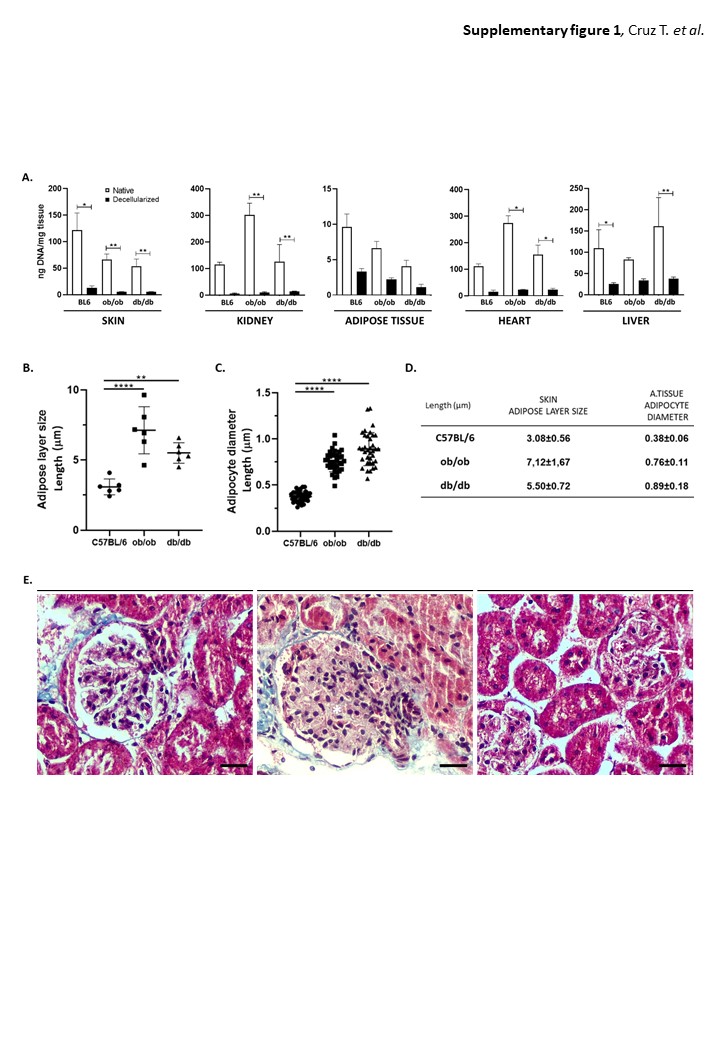

Supplement: Supplementary file 1 [file biomedicines-10-00057-s001.zip › Supplementary Figure S1_corrected.jpg]

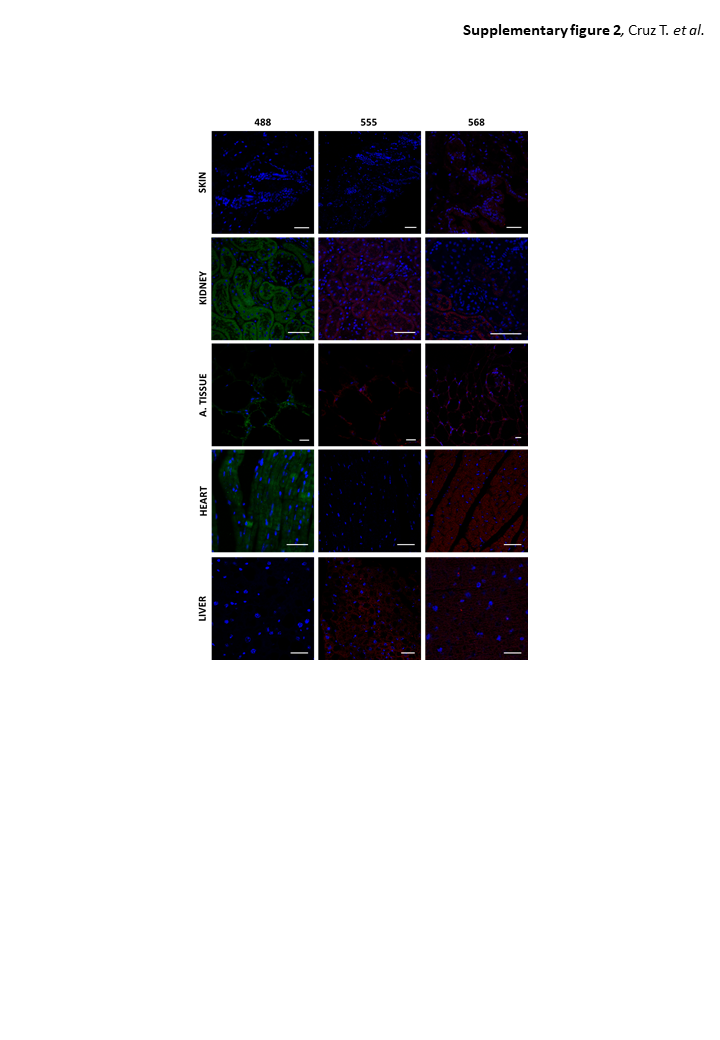

Supplement: Supplementary file 1 [file biomedicines-10-00057-s001.zip › Supplementary Figure S2.TIF]

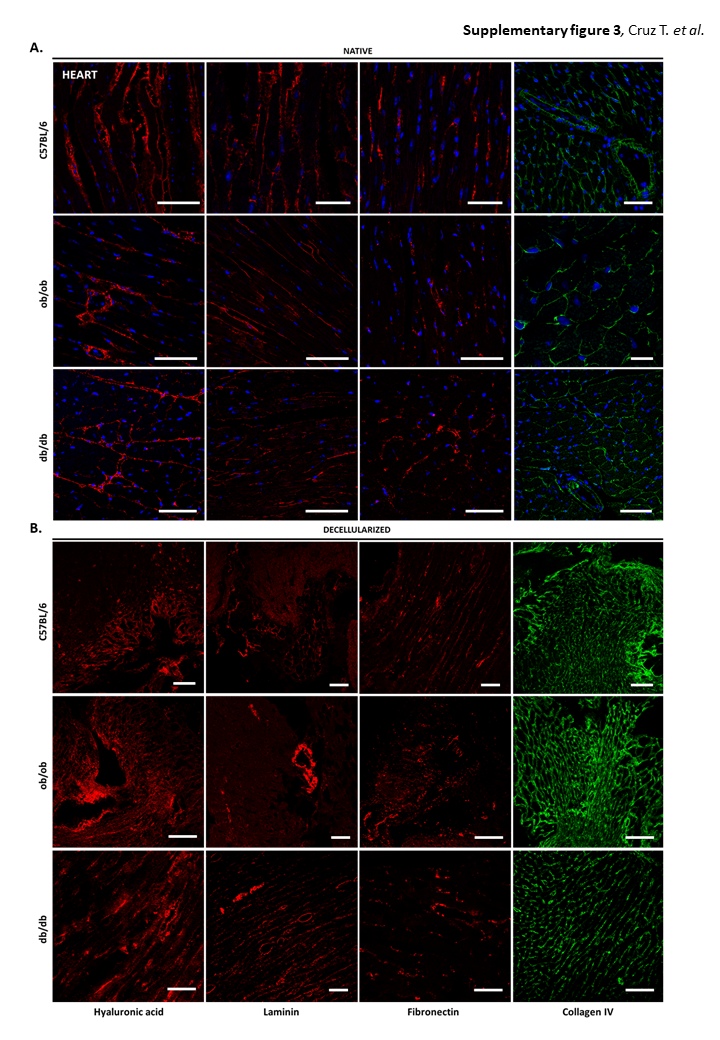

Supplement: Supplementary file 1 [file biomedicines-10-00057-s001.zip › Supplementary Figure S3.TIF]

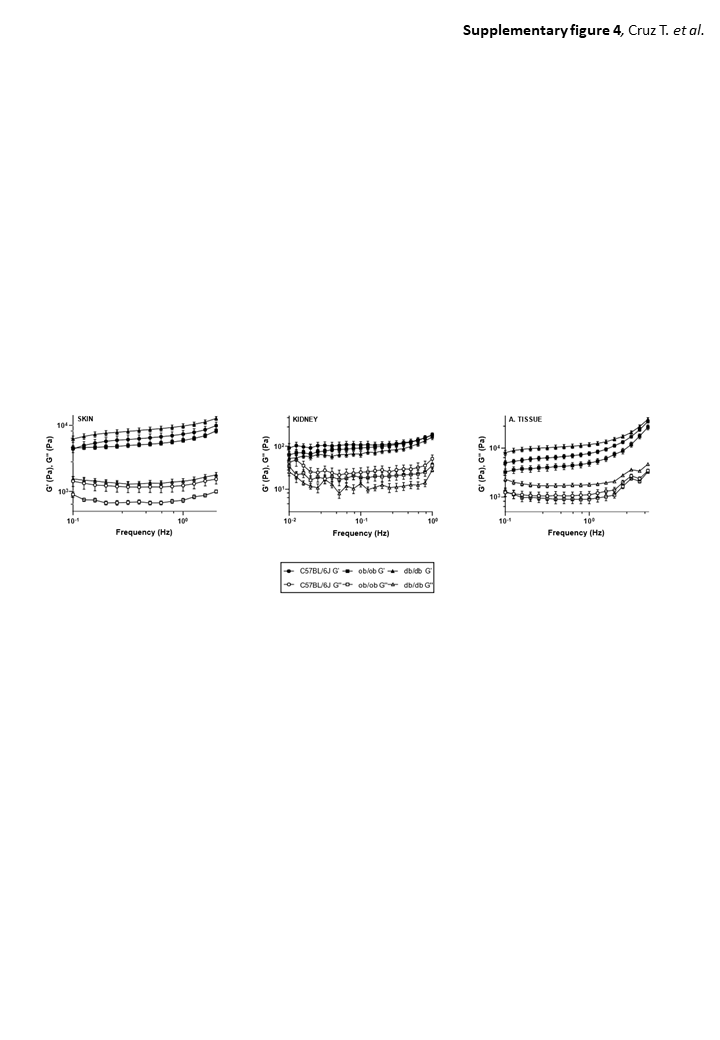

Supplement: Supplementary file 1 [file biomedicines-10-00057-s001.zip › Supplementary Figure S4.TIF]
